# Prevalence of Occupational Stress Among School Teachers Working in Ernakulam, Kerala, India

**DOI:** 10.7759/cureus.111743

**Published:** 2026-06-29

**Authors:** Shreyansh Bhushan, Shriya S Pillai, Sneha Daga, Shrinidhi Sivadhasan, Shreya Shrikant Jagtap, Shwetha Sajith, Srinidhi Chandra, Swetha Medammal, Sibin Selvam, Shyam Sundhar MK, Parvathi Sureshkumar, Shifa Habeeb, Brilly M Rose, Navami Sasidharan

**Affiliations:** 1 Community Medicine, Amrita School of Medicine, Amrita Institute of Medical Sciences, Amrita Vishwa Vidyapeetham, Ernakulam, IND; 2 Medicine, Amrita School of Medicine, Amrita Institute of Medical Sciences, Amrita Vishwa Vidyapeetham, Ernakulam, IND

**Keywords:** blood pressure, kerala, : occupational stress, teachers, workload

## Abstract

Background

Occupational stress is a major public health concern with significant implications for mental and physical health. Teachers are particularly vulnerable due to sustained emotional demands, workload pressures, and administrative responsibilities. Evidence regarding occupational stress among school teachers in Kerala remains limited.

Objectives

To assess the prevalence of occupational stress among teachers working in selected private schools in Ernakulam district, Kerala, and to identify sociodemographic, occupational, lifestyle, and clinical factors associated with occupational stress.

Methods

A cross-sectional study was conducted among teachers working in selected private schools in Ernakulam district, Kerala. Teachers employed for at least one month were included. Data were collected using a semi-structured questionnaire covering sociodemographic, occupational, lifestyle, and clinical variables. Occupational stress was assessed using the validated Teachers' Occupational Stress Scale (TOSS). Stress levels were categorized as low, moderate, and high. Blood pressure and anthropometric measurements were recorded using standard procedures. Data were analyzed using SPSS version 21. Associations were assessed using the chi-square test, and multivariable logistic regression analysis was performed to identify factors independently associated with moderate-to-high occupational stress.

Results

A total of 111 teachers participated, of whom 98.2% were female and 52.3% were aged ≥42 years. Based on TOSS, 64.0% (95% CI: 55.1%-72.9%) had moderate stress, 29.7% (95% CI: 21.2%-38.2%) had low stress, and 6.3% (95% CI: 1.8%-10.8%) had high stress. Occupational stress was significantly associated with blood pressure status (p = 0.014), teaching experience (p = 0.015), weekly teaching hours (p < 0.001), and commuting distance (p < 0.001). Teachers with less than five years of experience and those handling fewer than 30 teaching hours per week had significantly higher odds of moderate-to-high occupational stress.

Conclusion

Moderate occupational stress was common among teachers working in selected private schools in Ernakulam district. Shorter teaching experience and fewer weekly teaching hours were independently associated with moderate-to-high occupational stress. Workplace interventions focusing on mentoring, workload management, and mental health support may help improve teacher well-being.

## Introduction

Stress is an inevitable component of modern life and is recognized as a key determinant of mental and physical health. The WHO defines stress as a state of mental or emotional strain resulting from demanding or adverse circumstances [1]. While a certain degree of stress may be adaptive, sustained exposure to stressors can exceed an individual’s coping capacity and lead to adverse psychological and physical outcomes. In occupational settings, stress arises when work demands exceed available resources, coping abilities, or social support [2].

At the global level, occupational stress has become a major public health concern. The WHO has recognized work-related stress as a key contributor to the global burden of disease, particularly due to its links with common mental disorders and chronic noncommunicable diseases [3]. Teachers are a professional group particularly vulnerable to occupational stress because of sustained emotional demands, role overload, time pressure, and growing administrative responsibilities [4]. Studies from Europe and Africa consistently report high levels of stress among teaching professionals [5,6].

Occupational stress among teachers has implications that extend beyond individual discomfort. At the personal level, prolonged stress is associated with fatigue, sleep disturbances, emotional exhaustion, anxiety, reduced concentration, and an increased risk of chronic conditions such as hypertension [4,7]. Persistent exposure to stress may impair coping ability, reduce motivation, and negatively affect overall well-being. At the organizational level, high stress among teachers contributes to absenteeism, reduced productivity, job dissatisfaction, poor classroom performance, and premature attrition from the profession, thereby affecting the overall quality and sustainability of the education system [4].

Despite Kerala being known for its high literacy rates and well-developed educational infrastructure, evidence regarding the magnitude of occupational stress and its associated factors among school teachers at the state level remains limited. Understanding these factors is essential for planning targeted workplace interventions and stress management strategies for teachers.

Therefore, the present study was undertaken to (i) assess the prevalence and severity of occupational stress among teachers working in selected private schools in Ernakulam district, Kerala, and (ii) identify the sociodemographic, occupational, lifestyle, and clinical factors associated with occupational stress in this population.

## Materials and methods

A cross-sectional study was conducted among teachers working in four selected private schools in Ernakulam district, Kerala, over a two-week period during March 2025. Four schools (two urban and two rural) were selected based on feasibility of access and administrative permission. All eligible teachers present during the study period were invited to participate. Teachers who had been working in the selected schools for at least one month were included, as this duration was considered sufficient for them to become familiar with the school environment, workload, and occupational stressors. Teachers who did not provide written informed consent were excluded. A total of 111 eligible teachers consented to participate, and there were no incomplete responses.

The sample size was calculated using the formula n = Z²pq/d², where p = 0.55 based on a previous study from Thalassery, Kerala [8], q = 1 - p, Z = 1.96 at a 95% confidence level, and relative precision (d) was taken as 20% of p. The minimum required sample size was 82. Purposive sampling was used to recruit participants from the four selected private schools. Data were collected using a self-administered, semi-structured questionnaire created in Google Forms to obtain information on socio-demographic characteristics, occupational details, lifestyle factors, and self-reported morbidities. Weekly teaching hours and commuting distance were categorized based on the distribution of the study population. Skipping meals referred to the self-reported omission of one or more regular meals due to teaching responsibilities. Participants completed the questionnaire independently under the supervision of the investigators during school hours.

Occupational stress was assessed using the Teachers’ Occupational Stress Scale (TOSS) [9], a copyrighted instrument published by the National Psychological Corporation, for which permission was obtained from the copyright holder before data collection. The instrument consists of 30 items distributed across five domains: workload, student misbehaviour, lack of professional recognition, lack of classroom resources, and poor colleague relations. Responses were recorded on a five-point Likert scale and scored using direct and reverse coding according to the instrument manual. Domain-wise scores were summed to obtain the total TOSS score, which was standardized using Z-scores and categorized into seven levels ranging from extremely low to extremely high stress. For descriptive analysis, stress was categorized as low, moderate, and high according to the instrument manual. For association and multivariable analyses, the moderate and high stress categories were combined and analysed as moderate-to-high occupational stress to improve model stability.

Ethical clearance was obtained from the Institutional Ethics Committee (ECAMS-AIMS-2024-155), and written informed consent was obtained from all participants prior to data collection. Height and weight were measured using standard procedures to calculate BMI, which was classified according to the WHO Asia-Pacific classification for obesity in Asian populations [10]. Blood pressure was measured using an automatic sphygmomanometer after participants had rested for at least five minutes in the sitting position and was categorized as normal, prehypertensive, or hypertensive according to the Eighth Joint National Committee (JNC 8) criteria [11].

The collected data were entered into Microsoft Excel and analysed using SPSS version 21. Continuous variables were summarized as mean and standard deviation, while categorical variables were expressed as frequency and percentage. The Chi-square test or Fisher's exact test, as appropriate, was used to assess associations between occupational stress and selected sociodemographic, occupational, lifestyle, and clinical variables. Variables with p < 0.20 on bivariate analysis were entered into a multivariable logistic regression model using the backward likelihood ratio method. Adjusted odds ratios (AORs) with 95% CIs were calculated, and a p-value <0.05 was considered statistically significant.

## Results

A total of 111 school teachers participated in the study. The majority, 58 (52.3%), were aged 42 years or older, and most were female (109 (98.2%)). Regarding socioeconomic status, 96 (86.5%) belonged to the Above Poverty Line (APL) category. In terms of educational qualifications, 39 (35.1%) were graduates, while 72 (64.9%) were postgraduates. Most participants were married (98 (88.3%)), with smaller proportions being unmarried (11 (9.9%)) or separated (2 (1.8%)). A total of 66 (59.5%) teachers were employed in rural schools.

Regarding the nutritional and clinical profile, 24 (21.6%) teachers had a normal BMI, 84 (75.7%) were overweight or obese, and 3 (2.7%) were underweight. According to the Eighth Joint National Committee (JNC 8) criteria, 48 (43.2%) participants had normal blood pressure, 48 (43.2%) had elevated blood pressure, and 15 (13.5%) were hypertensive.

Concerning work characteristics, 28 (25.2%) teachers had less than five years of teaching experience, while 83 (74.8%) had five years or more. Nearly half of the participants (48 (43.2%)) handled fewer than 30 hours of classes per week, 40 (36.0%) handled 30-36 hours, and 23 (20.7%) handled more than 36 hours per week. Regarding commuting distance, 48 (43.2%) lived within 5 km of their school, 32 (28.8%) lived 5-10 km away, and 31 (27.9%) lived more than 10 km from their workplace. In terms of lifestyle factors, 93 (83.8%) reported sleeping 5-7 hours per day, and 34 (30.6%) reported skipping meals due to teaching responsibilities.

**Figure 1:**
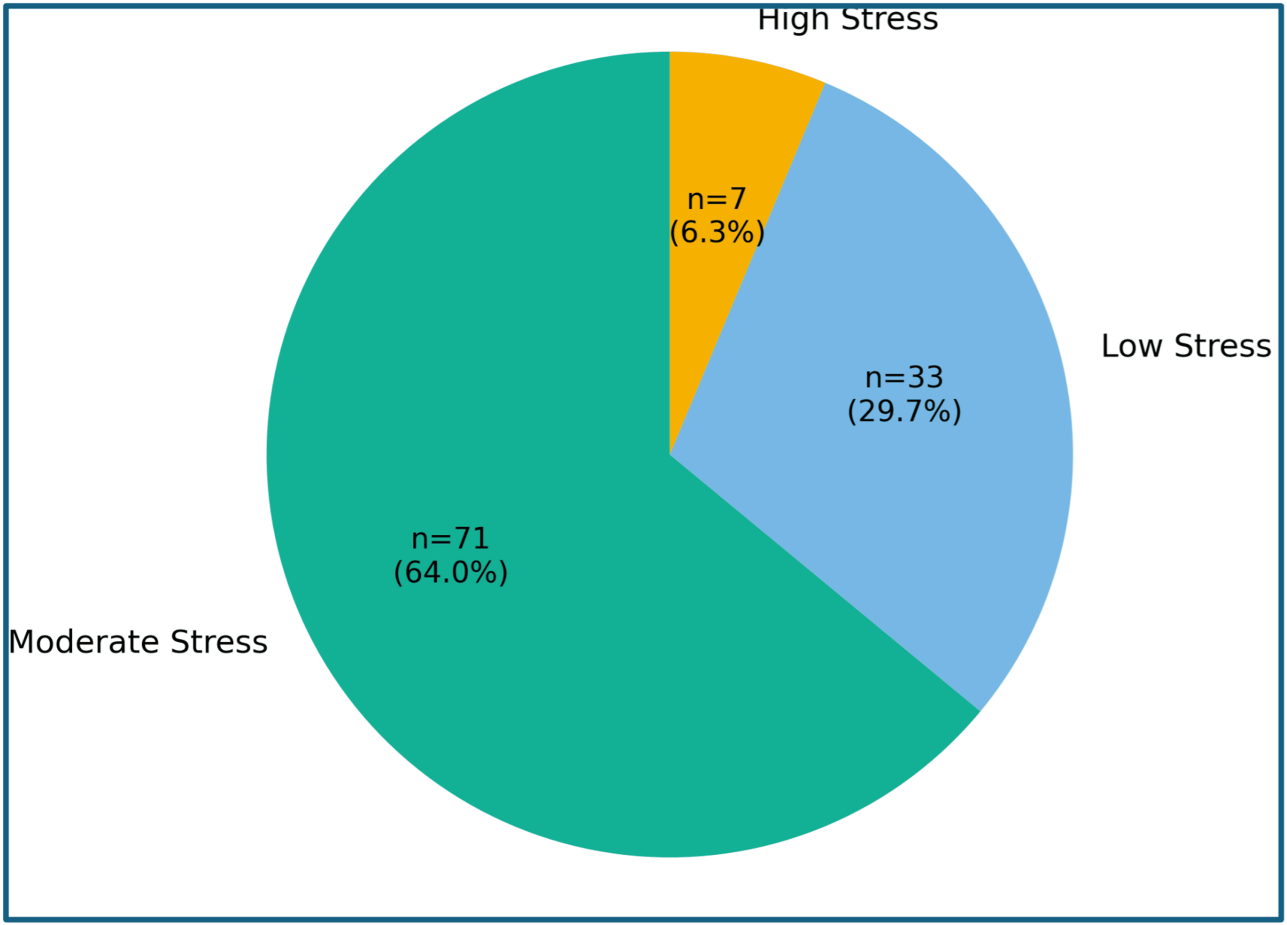
Distribution of occupational stress among study participants (n = 111). The pie chart depicts the distribution of occupational stress among school teachers as assessed using the Teachers' Occupational Stress Scale (TOSS). Moderate stress was the most common category (71 (64.0%)), followed by low stress (33 (29.7%)) and high stress (7 (6.3%)).

Based on the TOSS, the majority of participants (71 (64.0%; 95% CI: 55.1%-72.9%)) had moderate stress, followed by low stress (33 (29.7%; 95% CI: 21.2%-38.2%)). Only a small proportion (7 (6.3%; 95% CI: 1.8%-10.8%)) experienced high stress (Figure 1).

**Figure 2:**
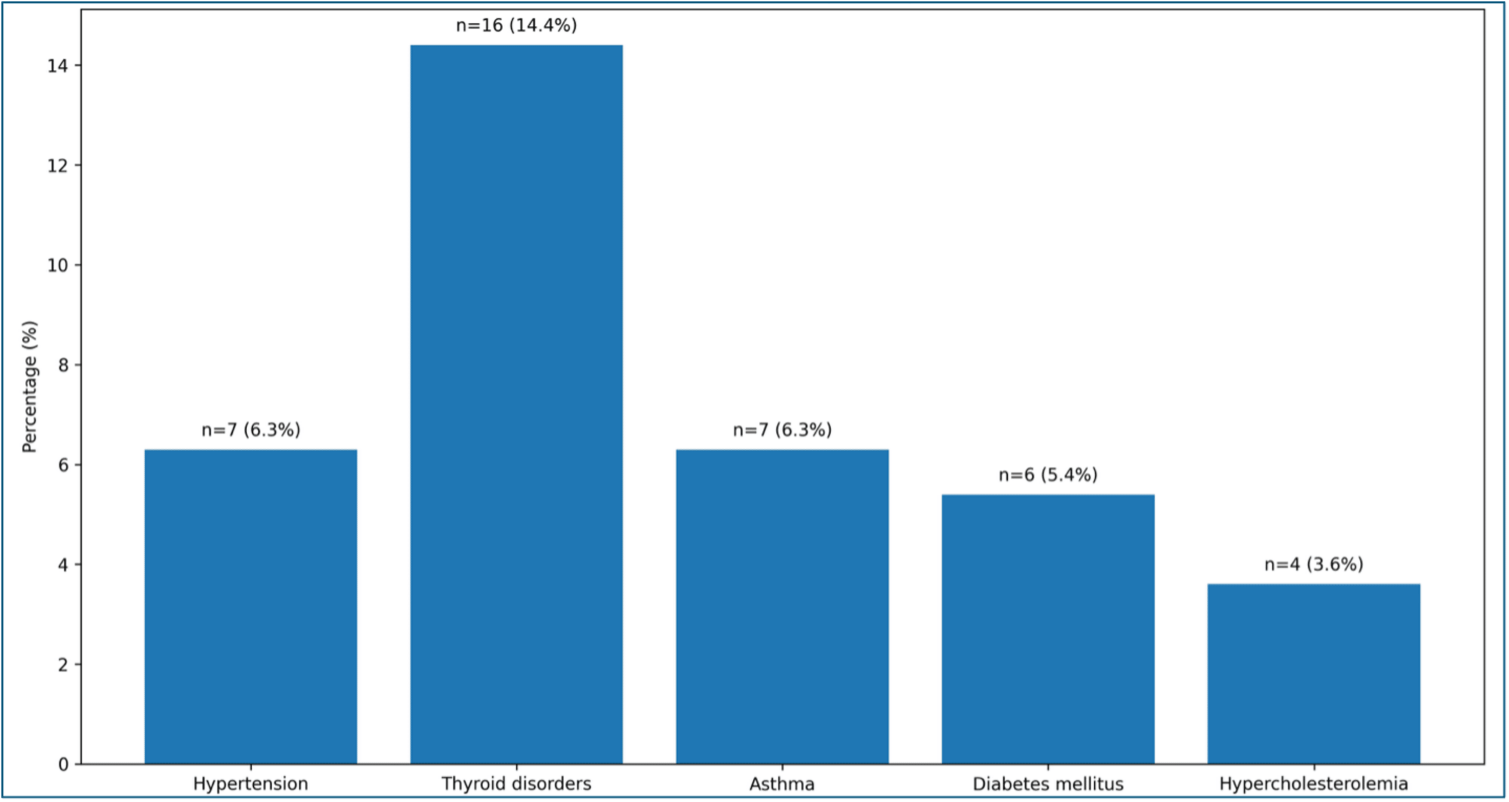
Distribution of self-reported morbidity among school teachers (n = 111). The bar chart depicts the distribution of selected self-reported chronic morbidities among school teachers. Thyroid disorders were the most commonly reported morbidity (16 (14.4%)), followed by hypertension (7 (6.3%)), asthma (7 (6.3%)), diabetes mellitus (6 (5.4%)), and hypercholesterolemia (4 (3.6%)).

With regard to self-reported morbidity status, 7 (6.3%) teachers reported hypertension, 16 (14.4%) reported thyroid disorders, 7 (6.3%) reported asthma, 6 (5.4%) reported diabetes mellitus, and 4 (3.6%) reported hypercholesterolemia, while the majority did not report any chronic illness (Figure 2).

For analysis, the moderate and high stress categories were combined and analysed as moderate-to-high occupational stress. On univariate analysis, occupational stress was found to be significantly associated with blood pressure status (p = 0.014), teaching experience (p = 0.015), number of teaching hours per week (p < 0.001), and distance between residence and school (p < 0.001).

Moderate-to-high occupational stress was more common among teachers with elevated or hypertensive blood pressure, less than five years of teaching experience, fewer than 30 teaching hours per week, and longer commuting distances. No statistically significant associations were found between occupational stress and age, gender, socioeconomic status, educational qualification, marital status, school location, body mass index, sleep duration, skipping meals due to classes, or the presence of comorbidities (Table 1).

**Table 1:** Association between sociodemographic, lifestyle, and clinical factors and occupational stress (TOSS) (n = 111). †Fisher’s exact test. APL: Above Poverty Line; BPL: Below Poverty Line; TOSS: Teachers' Occupational Stress Scale. Values are expressed as number (percentage). Occupational stress was dichotomized into low occupational stress and moderate-to-high occupational stress categories. The association between each variable and occupational stress was assessed using Pearson’s chi-square test. The χ² statistic is reported for variables for which the chi-square test was applied. †Fisher’s exact test was applied instead of the chi-square test when any expected cell count was less than 5; for these variables, the χ² statistic is not reported. A p-value <0.05 was considered statistically significant.

Variable	Category	Moderate-to-high occupational stress, n (%)	Low occupational stress, n (%)	p-value	Test statistic (χ²)
Age (years)	<42	40 (75.5)	13 (24.5)	0.252	0.88
	≥42	38 (65.5)	20 (34.5)		
Sex	Female	77 (70.6)	32 (29.4)	0.508†	–
	Male	1 (50.0)	1 (50.0)		
Socioeconomic status	APL	67 (69.8)	29 (30.2)	1†	–
	BPL	11 (73.3)	4 (26.7)		
Education	Graduation	26 (66.7)	13 (33.3)	0.541	0.16
	Postgraduate	52 (72.2)	20 (27.8)		
Location of school	Rural	43 (65.2)	23 (34.8)	0.153	1.48
	Urban	35 (77.8)	10 (22.2)		
Skipping meals due to classes	No	51 (66.2)	26 (33.8)	0.161	1.38
	Yes	27 (79.4)	7 (20.6)		

On multivariable logistic regression analysis using the backward likelihood ratio method, teaching experience and hours of classes per week were independently associated with moderate-to-high occupational stress. Teachers with less than five years of experience had significantly higher odds of moderate-to-high occupational stress than those with five or more years of experience (AOR = 7.65, 95% CI: 1.95-30.07, p = 0.004). Similarly, teachers handling fewer than 30 hours of classes per week had significantly higher odds of moderate-to-high occupational stress than those handling 30-36 hours per week (AOR = 9.36, 95% CI: 2.95-29.67, p < 0.001). Teaching more than 36 hours per week was not significantly associated with occupational stress (AOR = 0.97, 95% CI: 0.30-3.11, p = 0.966). The model demonstrated moderate explanatory power (Nagelkerke R² = 0.318) (Table 2).

**Table 2:** Multivariable logistic regression analysis of factors independently associated with moderate-to-high occupational stress (n = 111). AOR: Adjusted odds ratio; Ref: Reference category. Values are expressed as number (percentage). AORs with 95% CIs were obtained from binary logistic regression, with occupational stress dichotomized as low versus moderate-to-high occupational stress. The Wald χ² statistic (df = 1) was used to assess the significance of each predictor. Reference categories are denoted as “Ref.” A p-value <0.05 was considered statistically significant.

Variable	Category	Moderate-to-high occupational stress, n (%)	Low occupational stress, n (%)	AOR (95% CI)	p-value	Test statistic (Wald χ²)
Teaching experience (years)	<5	25 (89.3)	3 (10.7)	7.65 (1.95-30.07)	0.004	8.5
	≥5 (Ref)	53 (63.9)	30 (36.1)	1	-	-
Teaching hours per week	<30	43 (89.6)	5 (10.4)	9.36 (2.95-29.67)	<0.001	14.424
	30-36 (Ref)	21 (52.5)	19 (47.5)	1	-	-
	>36	14 (60.9)	9 (39.1)	0.97 (0.30-3.11)	0.966	0.003

## Discussion

The present study found that moderate occupational stress was the most common level of stress among teachers working in selected private schools in Ernakulam district, with 64.0% of participants experiencing moderate stress, 29.7% low stress, and 6.3% high stress. A similar predominance of moderate stress (76.2%) was reported by Ngente L and Hnamte L among higher secondary school teachers in Mizoram [12]. Christian DS et al. from Ahmedabad also observed that most teachers experienced moderate occupational stress [13], suggesting that moderate stress is a common finding among teachers in different Indian settings.

In contrast, Desouky D and Allam H reported a higher proportion of teachers experiencing severe occupational stress in Egypt [14]. Differences in institutional workload, availability of support systems, educational policies, and sociocultural contexts may account for the variation in stress levels across studies.

Blood pressure status was significantly associated with occupational stress in the present study (p = 0.014), with moderate-to-high occupational stress being more common among teachers with elevated or hypertensive blood pressure. Similar findings were reported by Chetia D et al. in Assam [7], who observed an association between occupational stress and hypertension among teachers. Although chronic stress has been linked to sustained sympathetic activation and adverse cardiovascular outcomes, the cross-sectional design of the present study precludes causal inference.

Teaching experience was independently associated with moderate-to-high occupational stress, with teachers having less than five years of experience demonstrating significantly higher odds than those with longer experience. Similar findings were reported by Jahan H and Sharma S [15], where less experienced teachers experienced greater occupational stress. Early-career teachers may encounter challenges related to classroom management, adaptation to professional responsibilities, and role adjustment, which could contribute to increased stress. However, Agai-Demjaha T et al. reported lower stress among less experienced teachers [6], indicating that this relationship may vary across institutional and sociocultural settings.

The number of teaching hours per week was also independently associated with occupational stress. Teachers handling fewer than 30 hours of classes per week had significantly higher odds of moderate-to-high occupational stress than those handling 30-36 hours. This finding differs from that of Matsushita M and Yamamura S in Japan [16], who reported higher stress among teachers with longer working hours. The observed association in the present study may reflect unmeasured factors such as administrative workload, probationary employment, non-teaching responsibilities, or job insecurity among teachers with fewer teaching hours. Therefore, this finding should be interpreted with caution.

Distance between residence and school was significantly associated with occupational stress, with moderate-to-high occupational stress being more common among teachers residing farther from their workplace. Longer commuting distances may contribute to fatigue, reduced recovery time, and increased work-related stress [16].

The present study has several strengths, including the use of a standardized and validated instrument to assess occupational stress and the inclusion of teachers from both urban and rural schools, enhancing contextual relevance. Assessment of clinical and lifestyle variables also enabled exploration of multiple factors associated with occupational stress. However, the cross-sectional design precludes causal inference, and purposive sampling from only four private schools limits the generalizability of the findings. The predominance of female participants further limits applicability to male teachers. In addition, self-reported information may have introduced reporting bias, and unmeasured factors such as administrative workload, job insecurity, and organizational support may have resulted in residual confounding. Despite these limitations, the study provides useful evidence on occupational stress among teachers working in selected private schools in Ernakulam district and may inform future workplace mental health interventions and larger multicentre studies.

## Conclusions

The present study found that moderate occupational stress was common among teachers working in selected private schools in Ernakulam district, Kerala. Teaching experience and the number of teaching hours per week were independently associated with moderate-to-high occupational stress, while blood pressure status and commuting distance showed significant associations on univariate analysis.

These findings underscore the importance of promoting teacher well-being through supportive workplace environments, mentoring programmes for early-career teachers, appropriate workload distribution, and regular screening for occupational stress and related health conditions. Given the cross-sectional design and purposive sampling, the findings should be interpreted as associations rather than causal relationships. Further multicentre studies with representative sampling are warranted to confirm these findings and inform occupational mental health strategies for teachers.
